# Motion Vector Extrapolation for Video Object Detection

**DOI:** 10.3390/jimaging9070132

**Published:** 2023-06-29

**Authors:** Julian True, Naimul Khan

**Affiliations:** Department of Electrical, Computer and Biomedical Engineering, Toronto Metropolitan University, Toronto, ON M5B 2K3, Canada

**Keywords:** object detection, video, convolutional neural network, motion vector, optical flow

## Abstract

Despite the continued successes of computationally efficient deep neural network architectures for video object detection, performance continually arrives at the great trilemma of speed versus accuracy versus computational resources (pick two). Current attempts to exploit temporal information in video data to overcome this trilemma are bottlenecked by the state of the art in object detection models. This work presents motion vector extrapolation (MOVEX), a technique which performs video object detection through the use of off-the-shelf object detectors alongside existing optical flow-based motion estimation techniques in parallel. This work demonstrates that this approach significantly reduces the baseline latency of any given object detector without sacrificing accuracy performance. Further latency reductions up to 24 times lower than the original latency can be achieved with minimal accuracy loss. MOVEX enables low-latency video object detection on common CPU-based systems, thus allowing for high-performance video object detection beyond the domain of GPU computing.

## 1. Introduction

Object detection has seen significant progress over the last several years [1,2,3,4,5,6,7]. Each new iteration or approach promises higher accuracy at the cost of more inference latency or less latency at the cost of lower accuracy when compared with the high-latency counterparts. Latency occurs in object detection systems due to the high amount of computations required across the entire image for detecting objects. This is compounded by the fact that many object detection application areas (e.g., surveillance and self-driving) have incredibly strict latency requirements [8]. Computing companies are in an arms race to provide hardware that offers the capability to use high-accuracy models at low latencies, but at best, this introduces a dependency on off-the-shelf GPUs and, at worst, a dependency on expensive niche co-processors. Despite the progress made, the trilemma persists; there is not a silver bullet to address the three constraints of accuracy, latency, and cost simultaneously.

Optical flow has also had recent performance gains through the use of CNN architectures [9,10,11]. Through these methods and GPU hardware acceleration, dense optical flow techniques have become faster and more accurate over the last several years.

It is well established that image content varies slowly in video data, as following the same object over time can be viewed as a different task altogether compared with finding unique objects in every frame. Though there have been attempts to exploit this temporal information redundancy through feature propagation based on optical flow methods in the past, performance remains bottlenecked by the latency associated with a CNN inference [12].

This work proposes MOVEX, an online real-time method of object detection via video. Through the combination of an arbitrary off-the-shelf object detection deep neural network (DNN) with a coarse approximation of optical flow and an optimistic sparse detection propagation parallelism strategy, we demonstrate that fast, accurate, and computationally inexpensive video object detection can be achieved. Furthermore, this work demonstrates the capability to accelerate object detectors up to 24 times their original performance with minimal (<1.0 AP) accuracy degradation. Since MOVEX does not require the use of a GPU, it enables models typically limited to the realm of GPU computing to be used on commodity CPU hardware with less inference latency than that found in GPU implementations of the same model.

This work contributes the following:

1. A novel parallelism-based approach for incorporating motion vector data with CNN object detection, bypassing the existing bottleneck associated with high-latency DNN inferencing currently present in existing single-threaded motion-based detection and tracking methodologies;
2. A comparative analysis demonstrating that motion vectors stored as part of the video encoding, when utilized as a coarse approximation to optical flow, can be effective for frame-by-frame motion prediction while maintaining backward compatibility for current state-of-the-art dense optical flow methods such as FlowNet2.0 [10] with this methodology;
3. A comprehensive evaluation across multiple datasets that demonstrates the efficacy of the approach in reducing object detection latency in video while quantifying the accuracy impacts. (Sample characteristic curves are provided for understanding the trade-off between latency and accuracy.)

The code has been made available at https://github.com/juliantrue/movex, accessed on 11 May 2023.

## 2. Related Work

Before reviewing existing approaches, it is critical to make the distinction that MOVEX is an approach to object detection and not object tracking. Object detection is the problem of identifying all individual instances of an object class in a single frame, whereas object tracking is the problem of identifying all individual instances of an object class and consistently assigning each instance an ID across consecutive frames [13,14,15]. Despite the difference in problem definitions, there is a class of object tracking algorithms from which insights can be drawn known as tracking by detection [15]. Tracking by detection details a two-step tracking procedure:

- Compute object detection;
- Compute data association from the previously observed data.

To reiterate, MOVEX does not address the object tracking problem as it is a method of accelerating object detection in video, but there are similarities in the approaches taken by tracking-by-detection applications. Particular ideas pertaining to exploitation of temporal redundancies in video data for detection acceleration are explored but are not directly comparable as they solve different problems.

To organize the related works better, first, we discuss the image-based object detection approaches that are relevant to our work in Section 2.1. Then, we discuss works that have incorporated motion vectors into object tracking in Section 2.2. Finally, in Section 2.3, we discuss deep feature flow, which is the optical flow-based approach that we compare MOVEX with.

### 2.1. Object Detection Approaches

Object detection approaches can be divided into two types: two-stage detectors, which perform localization first and then classification, and one-stage detectors, which perform localization and classification simultaneously. Since the proposed approach can be incorporated into any existing object detector seamlessly, we elaborate upon and compare them against two popular methods, as described in the following sections.

#### 2.1.1. Faster R-CNN

Faster R-CNN is a two-step object detection architecture presented by Ren et al. that consists of feature extraction and region proposal followed by a classifier [3]. This method achieves state-of-the-art results in object detection tasks such as PASCAL VOC [16] and COCO [17]. The authors created a region proposal network, which is a fully convolutional network that acts as a trainable object proposal model, and it simultaneously provides class-agnostic objectness scores and bounds. These contributions significantly improved the runtime of the object detection model when compared with its predecessor Fast R-CNN [18].

#### 2.1.2. YOLOv4

Compared with Faster R-CNN, YOLOv4 is a more recent architecture in the object detection space. YOLOv4 breaks from its YOLO predecessors in the sense that it is the first YOLO model to implement an architecture inspired by two-stage detection architectures, being more similar to Faster R-CNN as opposed to the classic single-stage architecture, as proposed in the original YOLO paper [19]. Bochkovskiy et al. proposed several changes in this paper that lend to better accuracy with a faster runtime. Namely, they propose the use of CSPDarknet53 [20] in a two-stage detection scheme with region proposals generated by spatial pyramid pooling (SPP) [21] and a path aggregation network (PAN) [22]. The head for their object detector was the original YOLOv3 head network [2]. In addition to this architecture, the authors present a comprehensive analysis on the use of Bag of Freebies (BoF) and Bag of Specials (BoS). BoF refers to training methods that result in better accuracy without raising the cost of inference, and BoS refers to the use of post-processing techniques that increase the cost of inferencing slightly but significantly improve the performance of the object detector. YOLOv4 implements many of these BoF and BoS sets, for which the authors present strong evidence for use in their architecture [1].

### 2.2. Motion Vector-Based Object Tracking

Several pieces of object tracking research utilize motion vectors to accelerate their tracking performance [23,24,25]. The common approach among these works is interactions between an off-the-shelf object detection approach, such as Faster R-CNN [3] or YOLOv4 [1] explained above, and the motion vectors or motion predictions of each frame. Each approach computes a CNN inference as part of the object detection step at a sparse key frame and perturbs the detections by using the motion vectors through linear interpolation [23,24,25]. This approach yields increased the frame processing rates in all three of these approaches, ranging from 4.6 times for Tabani et al. [25] to 6 times for Liu et al. [24] in off-the-shelf hardware and 12 times in specialized hardware, such as an FPGA, for Ujiie et al. [23].

It is worth considering the claim of “real-time” performance made by these identified prior works. If a high-latency CNN inference is to be made every *k* frames, and fast interpolation computation is to be carried out for all other frames, then the average runtime will decrease. Yet, despite the average latency decreasing, claiming these approaches are “real time” is misleading, as the CNN inference latency will limit the instantaneous performance to its inference latency. When considering an approach to be real time, it is important to clarify that these approaches may be real time according to the average frame-rate but not actually real time when considered on a frame-by-frame basis due to the CNN latency bottleneck.

### 2.3. Deep Feature Flow

Video object detection is a superset of single-frame object detection, which incorporates temporal or inter-frame data from more than the current frame to improve the object detection performance.

This work builds on the problem formulation provided by Zhu et al. known as deep feature flow [12]. It frames video object detection as a two-step algorithm consisting of expensive feature extraction at sparse key frames and feature propagation for non-key frames. Through the use of a function, they coin sparse feature propagation [12]:

(1) $$f_{k}=W\left(f_{i},M_{i\to k},S_{i\to k}\right)$$

Equation (1) provides the ground work for propagating features forward from frame $I_{i}$ to frame $I_{k}$ through the use of the sparse feature propagation function W, which accepts as input the feature map from frame *i*, the 2D optical flow field $M_{i\to k}$, and a so-called scale field $S_{i\to k}$. The authors used a CNN-based method known as FlowNet [10] to estimate the flow $M_{i\to k}$ and added an extra channel to estimate the scale field $S_{i\to k}$. Additionally, they used a ResNet 50 and 101 network with the classification layers removed for the feature extractor backbone [26] and turned it into an object detector through the use of an R-FCN head network on top [12,27].

Through this work, Zhu et al. demonstrated that this approach is effective at reducing the average latencies associated with video object detection [12]. This technique, however, did not fully redress the high-latency operation of performing an inference with a CNN. Although inferencing less on a sequence of images does lower the average, it does not eliminate the necessity for a blocking high-latency inference every *k* frames.

## 3. Motion Vector Extrapolation (MOVEX)

The MOVEX technique involves modifying the deep feature flow approach to object detection in three ways:

1. Motion vectors stored as part of the video encoding are evaluated as a coarse approximation to optical flow while maintaining backward compatibility for dense optical flow methods such as FlowNet2.0 [10].
2. The sparse feature propagation function is reimagined as statistical aggregation followed by perturbation. This has the benefit of not requiring a GPU to perform pixel-wise computations for propagating features to the next frame, as required by the original technique [12].
3. A parallelism strategy building on the sparse feature propagation idea was implemented to remove the bottleneck of key frame computation present in the original sparse feature propagation approach. This method is called optimistic sparse detection propagation.

### 3.1. Coarse Optimal Flow Approximation

Modern video codecs such as VP9 and H.265, as well as older codecs such as H.264, encode video with intra-frame and inter-frame coding techniques. In order to reduce the entropy between successive frames, these video codecs implement a macroblock (MB) structure that allows for pixel patches to be translated within the frame before subsequently finding the difference between successive frames. These intra-frame translations that minimize the mean absolute difference (MAD) between image patches (and thus successive frames) are referred to as motion vectors (MVs) [28].

It is notable that these vectors do not specifically encode inter-frame object translations; despite their name, they encode the vector that minimizes the differences between successive macroblocks. However, it is often the case that they provide a reasonable approximation of such inter-frame object motion [29]. It is important to note that not all H.264 encodings are created equally. Lower-quality settings for such video codecs often yield poor motion vector representations while achieving their goal of minimizing the MAD in a lower search time or fewer vectors, if any. As such, the scope of motion vector encoding’s applicability to optical flow approximation remains limited to only higher-quality encodings.

Despite downstream applications attaining these artifacts or features for free, since they are pre-computed at encoding time, very few applications make use of them. When taking into account the quality considerations, H.264 motion vectors allow for extremely fast optical flow approximations.

In addition to the inherent computational savings associated with using pre-computed optical flow vector approximations, the encoded vectors are also sparse (one vector per macroblock), allowing for further computational savings if subsequent computational steps are considered. Performing pixel-level optical flow computation becomes expensive as the image resolution increases, and thus the necessity for GPU-based parallelism becomes apparent even at reduced resolutions, as shown in Table 1.

### 3.2. Optimistic Sparse Detection Propagation

The objective of optimistic sparse detection propagation is to accept a prior set of detections and perturb them according to the sparse detection propagation function (SDPF) W which, similar to the work of Zhu et al. [12], takes a 2D flow field ($M_{i\to k}$) (In our case, this will be the set of motion vectors.) as input, and rather than passing a set of features, our function accepts a prior set of object detection bounding boxes $D_{i}$. This difference allows our approach to be entirely model agnostic and thus not tied to one particular object detector:

(2) $$D_{k}=W\left(D_{i},M_{i\to k}\right)$$

The SDPF iterates over each detection $d_{j}$ in the set $D_{i}$ and applies an aggregation function ϕ to the enclosed flow vectors $m_{uv}$, resulting in a net flow vector to perturb the detection with. Each 2D flow field is stored in a temporary buffer B. A specific flow vector is defined as the difference in image coordinates from starting frame 1 to consecutive frame 2, resulting in a vector formatted as $m_{uv}=\left(u_{2}-u_{1},\;v_{2}-v_{1}\right)$ where *u* and *v* are the image coordinates:

(3) $$d_{k}=\phi \left(d_{j},m_{uv}\right)$$

**Figure 1:**
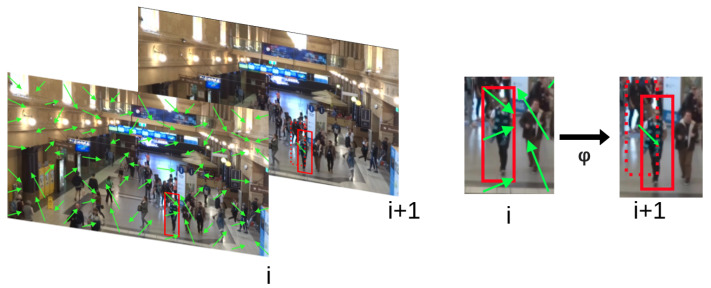
Visualization of utilizing motion vectors to propagate a prior set of detections from a source frame to a consecutive frame (Motion vectors are denoted with green arrows. Red squares denote the detection bounding boxes). The only motion vectors considered in the source frame *i* are those which fall within the area of the bounding box. The median perturbation of those motion vectors is computed and applied to the source bounding box in order to predict the bounding box in frame i+1.

In practice, the aggregation function is simply the mean or median in *x* and *y*, but more complicated aggregation functions that weigh areas of the detection more than others can be considered. Figure 1 depicts the role of the aggregation function in propagating detections from the current frame to a consecutive frame.

Since the aggregation function only considers local features, global frame movement information is not effectively captured. In early iterations, it was observed that this led to compounding errors in video streams with camera movement. In order to combat the observed compounding errors, a global motion compensation term was implemented to correct for such camera movements through aggregating over the entire frame’s motion vectors. Equation (4) provides the final equation utilized as the aggregation function, which includes both the aforementioned local detection motion aggregation as well as the global frame motion aggregation:

(4) $$d_{k}=\phi_{local}\left(d_{j},m_{uv}\right)+\phi_{global}\left(m_{uv}\right)$$

The SDPF requires a starting set of detections to propagate forward through the video. This particular set is known as the prior detection set, which is an estimate provided from a key frame inference. However, rather than evoke a computationally expensive and blocking DNN inference at a key frame, this inference is computed in parallel. The object detector runs in parallel with another worker which simply iteratively applies an SPDF following Equation (2) to the existing detection set at frame *i*.

Since there is no waiting for object detection to complete before proceeding, the process which iteratively applies the SDPF works several frames ahead of the object detector before receiving the computed detections. As such, there is a discrepancy of several frames between the current set of detections and the returned detections from the object detector. However, since the flow fields have been retained every frame in the flow vector buffer B, the detections received from the inferencing process are propagated forward through iteratively applying the SDPF on said buffer of flow vectors in order to update the prior ones. At the end of this update, the detections at the current frame incorporate the computed detection information from the DNN worker. Articulated another way, when new information is returned for a frame that has already passed, the stored flow fields are used to re-propagate the new detection set forward to the current frame. The buffer is emptied in this update to allow for new flow vectors to be added.

Since there is no scheduling for prior key frame updates based on the elapsed time or frame index, existing detections will continue to be propagated forward in time until the prior is updated with new information from the object detector. As such, the object detector latency does not directly contribute to the computation time of predicting detections at a frame *i*. However, as the object detector latency increases, more frames will have passed during the elapsed computation time and thus will fill the motion vector buffer B to a greater capacity. As B fills with more frame data, the cost of a prior update becomes larger due to the number of frames for which detections need to be propagated forward to arrive back at the current frame *i*.

As the object detector latency increases, the interplay between updating detections based on image content versus updating based on flow vectors becomes apparent. New detection targets can only be detected with the object detector, and thus higher latencies will ultimately determine the performance in applications that have targets which enter and exit the image frame quickly.

**Figure 2:**
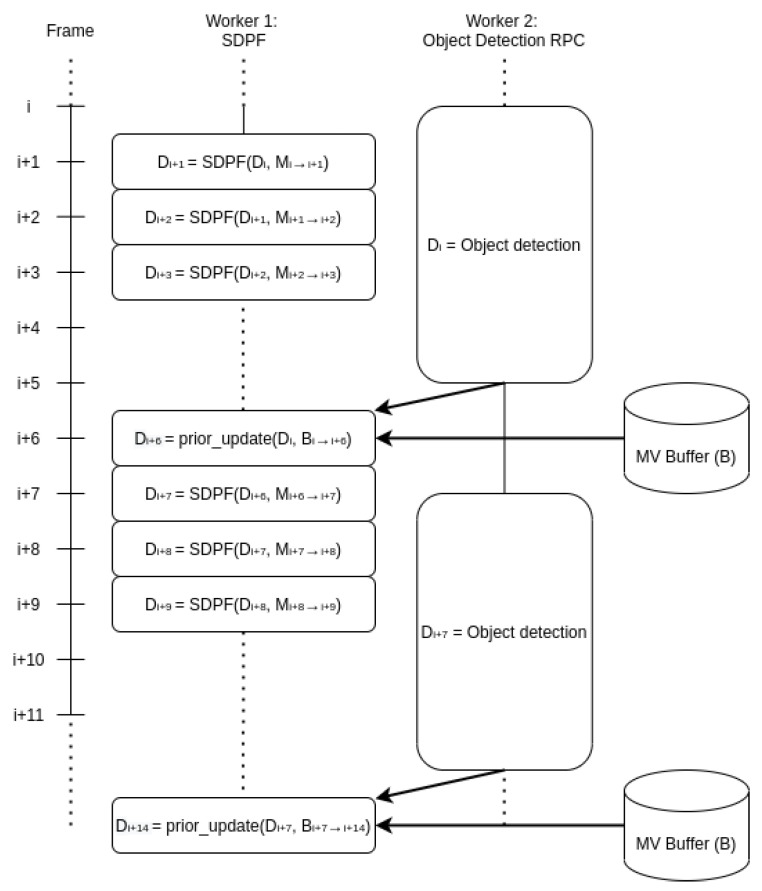
MOVEX implementation diagram.

This parallelism strategy is depicted in Figure 2, which highlights the interactions between the object detector and the detection propagation function. It is clear that Worker 1 applied the SDPF repetitively, which required significantly less single-threaded performance as its runtime was significantly faster than its object detection counterpart in Worker 2. Decoupling the object detection latency from the perturbation of existing information allows MOVEX to treat the SDPF as the rate-limiting step as opposed to object detection.

## 4. Experiments

### 4.1. Set-Up

In order to evaluate the capabilities of this approach, two critical metrics were considered: average precision (AP) and average detection latency. The datasets used to evaluate these metrics were the MOT20 and MOT16 datasets [14,30]. The reason for using these datasets was that the data in this case were taken directly from the video and maintained the temporal context between images, unlike other object detection datasets. The MOT challenge series of datasets that MOT20 and MOT16 are a part of are widely used for benchmarking throughout object tracking [15]. Through the combination of these two datasets, a representative evaluation of performance in various scenarios was able to be quantified. Datasets such as KITTI [31,32] were not considered, since they are primarily based on automotive data and will not form a representative evaluation of performance across domains.

MOT20 maintains a consistent camera location throughout each video sequence but has many detection targets per frame, making it challenging in that regard. Dendorfer et al. created this dataset specifically to create a benchmark for evaluating object detectors and trackers in “...very crowded scenes in which the density can reach values of 246 pedestrians per frame” [14]. As mentioned earlier, the videos of this dataset were all taken from a static camera at a high vantage point, and they range in time from 17 s up to 2 min and 12 s. The video was recorded at various locations such as a train station, a crowded plaza, and a crowded parking lot, possibly after an event of some sort. All videos in this dataset are at a 1080×1920 resolution except for one video sequence, which is 1174×880 [14].

MOT16 was evaluated as well, since it includes video sequences that contain a lot of global camera movement. The videos in this dataset are also far more diverse in terms of scenarios compared with the MOT20 dataset, including stationary video from a plaza, street, and inside a mall, as well as dynamic video taken from a handheld cell phone (presumably inside a mall), on the street, and video from a camera mounted on top of a large vehicle [30]. The videos in MOT16 are also of varying resolutions, as the lowest resolution is 640×480 and the highest is 1080×1920 [30]. The lengths of the videos were overall much shorter than those in the MOT20 dataset, as the shortest video was 17 s and the longest was 59 s [30].

MOT20 and MOT16 provide data in the form of JPEG-encoded images, which were converted to video data through the use of an H.264 encoder with encoding parameters given by the seqinfo.ini file for each evaluation sequence. Specifically, the libx264 open-source video codec was used with the built-in “slow” preset in order to ensure that the motion vectors generated were of good quality. The video was also generated such that there were no B frames computed. Through forcing this constraint on the codec, it ensured that the motion vectors generated frame by frame and referred to the forward differences only, thus maximizing the performance for the online nature of this technique.

When considering real-time scenarios, such as robotics applications, this evaluation approach will not present a full representation of the latency performance since the video under testing is encoded ahead of time. However, this latency can be accounted for in a particular application through measuring the latency of a specific encoder and adding it directly to the latency results we present. This self-imposed limitation on the latency results was due to the wide array of codec implementations in software and hardware available for use. In order to make our results pertinent to the most applications possible, we considered only the decoding time in the total latency and not the encoding time.

With each evaluation sequence, MOT20 provides detections from a Faster R-CNN [3] with ResNet 101 [26] backbone for evaluating tracking-by-detection applications. MOT16 also provides detections, but they are not from a Faster R-CNN model. For consistency, detections from a Faster R-CNN model provided by Yu et al. were used [33] for this dataset. Each model was trained solely on the MOT20 or MOT16 dataset. They also provided the evaluation kit for calculating the metrics of interest, such as the AP [14,30].

A mock RPC with adjustable latency was developed to serve these detections as if it came from a live RPC object detector. Through this method, it was possible to simulate the same model as if it were running as a remote process with variable latency. If one were to set the simulated latency of this mock RPC to zero, then every output detection would be the same as the provided detections. However, if the latency were to be increased, then frames would need to be extrapolated with MOVEX in order to remain as close to real time as possible. The simulated latency of the object detector was increased in order to characterize the relationship between the AP and output frame rate.

The aggregation function ϕ was implemented as a simple two-dimensional median calculation.

All evaluations were conducted on an Intel i7-8700K CPU (3.70 GHz) with an Nvidia GTX 1080Ti 12 GB GPU. Tests marked with “CPU” were evaluated solely on the CPU without exposing GPU capabilities to the test; otherwise, the GPU was used for evaluation.

### 4.2. Evaluations

We evaluated four key areas regarding this technique:

1. Object detector latency versus accuracy: we show the latency decrease versus the accuracy characteristics of the technique.
2. H.264 motion vectors and flowNet2.0: we show the performance implications of using H.264 motion vectors as opposed to a state-of-the-art method such as FlowNet2.0 [9].
3. High-resolution versus low-resolution object detection models: we show the viability of increasing the accuracy while decreasing the latency through the use of higher-input resolution models.
4. Object detection model inference on a CPU versus a GPU: we show the impacts of running the object detection model on a CPU instead of a GPU.

These four areas would be evaluated through AB testing of a model with and without the attribute of interest.

An ablation study was also conducted in order to further understand the contributions of each individual component in the MOVEX algorithm. The following elements of the MOVEX technique were ablated:

1. Global frame compensation was removed from the SPDF so that only the motion vectors located inside a given detection were considered in the propagating detections. We expected that through removing this component, the performance on the MOT20 dataset would remain roughly the same as there was very little camera movement. However, we expected that the performance on the MOT16 dataset would fall since there was a significant amount of camera movement in half the video sequences.
2. Multi-processing was removed from the optimistic SDPF and was replaced with a blocking inference every $k^{th}$ frame, which mimics the approach of Zhu et al. to detection propagation and other state-of-the-art approaches [12,23,24,25].
3. The aggregation function was replaced with a center sample approach to aggregating motion vectors. Rather than considering all motion vectors contained in a detection, only the center-most motion vector was considered. This test was performed in order to assess the impact of aggregation on the performance of the detection propagation function.
4. Detection propagation based on motion vectors was removed entirely, and instead, individual detections were persisted in place until a new detection was received, hence demonstrating the efficacy of the SPDF in its entirety. It was our expectation to see the performance fall significantly in the MOT16 dataset with this ablation, as that dataset has a great deal of variation from frame to frame. We also expected a less significant yet still large drop in accuracy in the MOT20 dataset. The reason we expected this is due to the sheer volume of the detection targets per frame moving in many different directions, as well as the associated occlusions.
5. The encoding quality was varied from high- to low-quality H.264 encodings as defined by the FFMPEG presets for the H.264 codec [34]. Not every application is able to use the specific encoding described in our approach, and as such, we quantified the effects the encoding quality had on MOVEX based on said common FFMPEG presets.

## 5. Results and Discussion

### 5.1. Object Detector Latency versus Accuracy and Overall Latency

**Figure 3:**
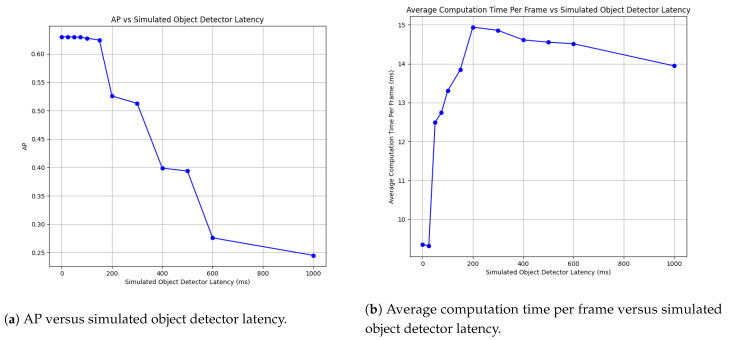
Simulated object detector latency versus AP (**a**) and average computation time for the proposed Movex approach on the MOT20 dataset (**b**). As the simulated object detector latency increased, the AP decreased, but the decrease only became apparent after increasing the latency to 200 ms. For reference, a data point with 0 ms of latency was included to depict the original performance of the object detector detections provided with the MOT20 dataset. The average computation time per frame rose as the object detector latency increased, and the prior update step became incredibly time consuming due to the size of the buffer accumulated during the object detector inference time.

As shown in Figure 3, the AP decreased as the simulated latency increased. Since the extrapolation step can only predict movements from existing detections, when new objects entered the scene, they were initially not detected and as such, driving down the AP. This has implications on types of objects tracked. If objects in the video are entering and exiting the frame on a relatively slow basis, than a high latency detector may be used with confidence. However, if objects are entering and exiting the frame at a rate much higher than the object detector latency, then the detection AP performance will fall.

**Figure 4:**
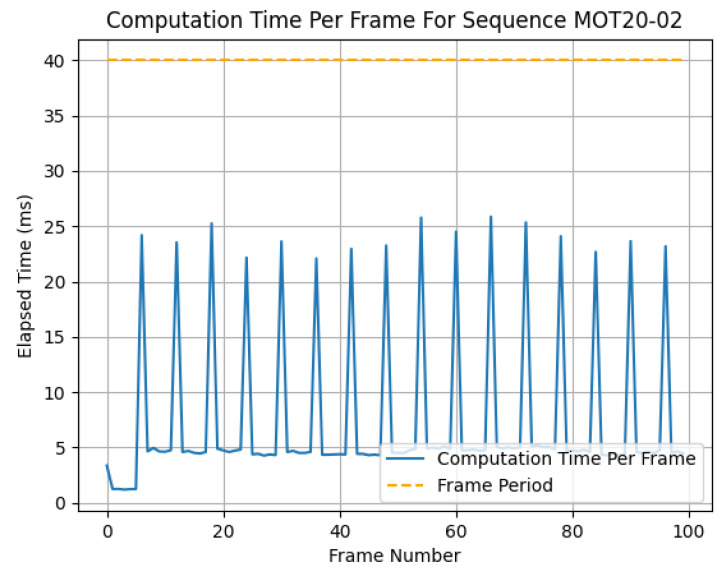
Latency versus frame number for a sample trace taken from the MOT20 dataset and the total computation time for first 100 frames of the sequence MOT20-02 using an object detector with 200 ms latency. The dashed line depicts the threshold considered real time for this particular sequence with a frame rate of 25 fps.

Additionally, in Figure 3, the average computation time per frame is given in relation to the object detector latency. The highest average computation time per frame occurred at an object detector latency of 200 ms, which indicates that the proportion of computation between prior updates and the looks ahead was dominated by the cost of prior updates. Figure 4 further demonstrates this point in the spikes in computation time for each prior update step.

Shown in Figure 4 is the output latency per frame for MOVEX when predicting detections. In order for this technique to be considered real time, it must compute the results before receiving the next input. This threshold is denoted by $\frac{1}{frame-rate}$ for a sample MOT20 sequence shown on the graph. The spikes in latency are the points in the sequence in which a prior update occurred. These peaks in latency represent the lowest possible latency for which MOVEX could be considered real time. In this case, approximately 26 ms (corresponding to a frame rate of 38.46 fps) was the fastest real-time update rate possible with an object detector latency of 200 ms.

Additionally, in Table 2, we show the results of the MOT16 evaluations. They are largely consistent with the results obtained from the MOT20 evaluations, with the exception of two notable entries. The third row and last rows of the table depict a much larger drop in AP in contrast to the MOT20 evaluations. Through observing the detection data overlaid on the video, the drop in performance on this dataset appears to be due to the challenging nature of the video sequences. Observationally, the significant increase in camera movement as well as varying camera perspectives caused many more instances of detections not forming a tight bound with their respective targets.

**Figure 5:**
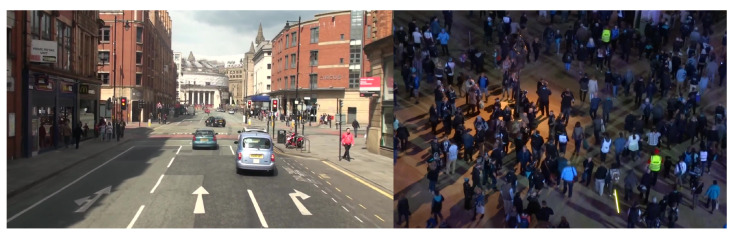
Sample frames from MOT16 dataset (**left**) and MOT20 dataset (**right**). These samples represent the different types of challenges inherent to the datasets. MOT16 is composed of moving cameras with a moderate number of people, while MOT20 is composed of largely static cameras focusing on large clusters of people.

As shown in Figure 5, the MOT20 dataset derives its difficulty from the sheer number of targets to detect, whereas MOT16 derives its difficulty from the challenging scenarios in which the videos were taken. We can conclude that these challenging, high camera movement scenarios presented in MOT16 result in a larger accuracy difference in higher latency object detectors. The subsequent sections present the results from MOT20 exclusively, since it was the most recent dataset in the MOTChallenge series at the time of writing and the MOT16 results corroborated the conclusions.

### 5.2. H.264 Motion Vectors and FlowNet2.0

When examining the performance results in Table 2, the latency of the original Faster R-CNN model was reduced by a factor of 9.52 times when run with MOVEX using the H.264 MVs. However, when using FlowNet2 as the source for the motion vectors, performance was greatly impacted, presenting a 1.97 increase in latency. The FlowNet2-s model was used to compute the optical flow, which claimed to have a runtime of approximately 7 ms on a GTX 1080Ti [9], but the performance when running it was nowhere near this, as the model’s forward passes were routinely reaching 80 ms. One reason for this discrepancy may be due the increased image resolutions of the images in the MOT20 dataset compared with the images the authors used for demonstration originally. The images in MOT20 all have a 1920×1080 resolution, while the images in KITTI 2015 have a 1248×384 resolution [31,32], and the Sintel dataset images’ resolution is 1024×436 [35]. This resolution difference resulted in a decrease of approximately four times in the image pixels when compared with the MOT20 data and thus could have lead to the latency increases observed. Shifting the focus to the AP differences between the three evaluated Faster R-CNN models demonstrates that the use of MOVEX decreased the AP of the baseline model by approximately 0.5 when using H.264 MVs but only led to a decrease of 0.1 when using the FlowNet2.0 model.

### 5.3. High-Resolution versus Low-Resolution Object Detection Models

The effect of increasing the input resolution for CNN object detectors is known to be increased accuracy. As shown in Table 2, YOLOv4 trained on the COCO dataset [17] was compared against itself at two different resolutions, 416×416 and 960×960, resulting in AP values of 26.1% (row 6) and 40.5% (row 4), respectively. This confirms the relationship between the input resolution and accuracy but also demonstrates an opportunity for improvement through the use of MOVEX. Consider that when using the higher resolution model with MOVEX (row 5), the AP dropped by a mere 0.1, yet the latency fell below that of the low resolution model (11.73 ms and 26.34 ms in row 5 versus row 6, respectively). This caries the implication that through using MOVEX, it is possible to achieve significantly more accurate object detection results for a given application latency budget through the use of a high-resolution model.

### 5.4. Object Detection Model Inference on the CPU versus the GPU

It is well known by practitioners that hardware accelerators such as GPUs or TPUs are needed to achieve low-latency computation with CNNs. This point is further articulated by the latency data point given in row 7 of Table 2, running the YOLOv4 model with an input resolution of 416×416 on a CPU. This yielded a latency of 190.41 ms, which is far too large for any real-time application. When using MOVEX in conjunction with this model, as shown in row 8 of Table 2, the latency fell below that of the original GPU computation latency, resulting in a latency reduction of 23.92 times while the AP only dropped by 0.4 when compared with the original model.

GPU computational resources are orders of magnitude more expensive than standard CPU-based systems. Employing MOVEX in systems looking to perform object detection on video data would lead to large cost savings by switching from GPU- to CPU-focused computing. Furthermore, emerging applications in edge computing where the cost, space, and computing capabilities are typically limited would greatly benefit from using this technique, since modern GPU-centered computing often clashes directly with these constraints.

### 5.5. Ablation Study

#### 5.5.1. Global Frame Compensation

The first ablation that was made was the removal of the global movement compensation mechanism. We present the results of this ablation in Table 3, which demonstrate that this step did not add significant latency to the process, since the latency was reduced by less than 1 ms in both datasets once removed. Additionally, since the AP decreased by 4.4 and 4.1 in both the MOT20 and MOT16 datasets, respectively, it is demonstrated that this global statistic is an important factor in the performance of the MOVEX technique.

#### 5.5.2. Multi-Processing

Table 4 denotes the results of inferencing every $k^{th}$ frame, such as in [12,23,24,25], versus the proposed multi-processing-based approach. The values of 5 and 10 in evaluation for *k* were compared.

It is shown in Table 4 that using this single-process approach resulted in significantly higher latencies than those for the proposed method and also resulted in substantial losses in accuracy in the cases where 10 was substituted for *k*.

#### 5.5.3. Aggregation Function

The impact of the aggregation function was ablated through the use of sampling one motion vector contained in the target detection, rather than combining all motion vectors from within said detection. The expectation from aggregating more information into the estimate of the detection target’s inter-frame motion would be that a more accurate propagated detection would be calculated. Thus, sampling the same location in the detection consistently would lead to a biased estimate and therefore a less accurate result.

The center of the motion vectors composed in the detection was found through computing the centroid of motion vectors given in Equation (5), where *n* is the number of motion vectors contained in the width of the detection and *m* is the number contained in the height, while *x* and *y* are the image coordinates of the starting point of said motion vectors:

(5) $$c=(\frac{1}{n}\sum_{i=1}^{n}x_{i},\;\frac{1}{m}\sum_{i=1}^{m}y_{i})$$

Since the centroid of the motion vectors does not necessarily correspond to a specific motion vector, the motion vector minimizing the Euclidean distance to the centroid was sampled as given by the following equation. Table 5 shows the results of this test:

(6) $$mv_{uv}=min_{xy}\sqrt{{\left(c_{x}-x\right)}^{2}+{\left(c_{y}-y\right)}^{2}}$$

As is common among other ablations, the removal of this feature appeared to yield very minute differences in the MOT20 data due to the slow varying nature of the data. However, the fast varying data of MOT16 were quite sensitive to this change, and omitting this resulted in a 0.6 drop in the AP.

#### 5.5.4. Detection Propagation

In the previous ablation, the aggregation function was replaced with the center sample approach to evaluate the impact of aggregation. This ablation extended that idea a step further through ablation of the entire SPDF in order to evaluate its performance impact. This was accomplished through replacing the aggregation function with a passthrough function which merely returned the detections it received as input. No aggregation or propagation was performed on the set of detections between frames; rather, the existing set of detections merely persisted from frame to frame. The detections in this ablation were only updated upon the object detector completing a computation and remained visually frozen in place across subsequent frames until another detection inference was completed.

In Table 6, it is shown that for MOT20, there was minimal impact from removing the detection propagation, while for MOT16, there was a significant drop in the AP from 54.6 to 50.1%, a drop of 4.5 in the AP. Through observing the video in the two datasets, it is clear that the high camera movement data, which were found in the MOT16 dataset, caused large drop-offs in accuracy when not accounted for by this technique. The order of magnitude of the drop in latency, however, is an interesting benefit to removing inter-frame detection propagation altogether. For video data that are sufficiently similar between DNN inferences, such as the surveillance camera-style footage of slow-walking pedestrians found throughout MOT20, it may be a worthwhile trade-off for some to deliberately omit the detection propagation step entirely to gain the order of magnitude for the latency decrease. However, it is unlikely that this would be a viable option for higher latency object detectors, since this approach would result in more frames with detections persisting in place, leading to poor accuracy.

#### 5.5.5. Encoding Quality

As MOVEX is dependent on the motion vectors present in the encoded video, it is a worthy question to ask what the impact on the encoding quality is on the performance of MOVEX. We can envision MOVEX being utilized in applications where the video is being streamed with a particular encoding quality, depending on the availability of resources. Therefore, we need to know what effect the encoding quality has on the performance of MOVEX. To answer this question, we varied the encoding quality presets inside the H.264 codec by using FFMPEG presets [34]. Specifically, we made use of the slow, medium, and fast presets, which correspond to the encoding qualities of high, medium, and low. This is by no means an exhaustive evaluation of specific variations arising from encoding variable changes, but it does provide a general overview of performance against the most common presets.

As shown in Table 7, for MOT20, there was a mere 0.1 change in the AP upon decreasing to the lower-quality encodings. However, for MOT16, there was a drop from the original performance of 54.6 to 50.9% when comparing the high quality to the medium and low qualities.

## 6. Conclusions

We presented MOVEX, a technique that can be applied to an arbitrary off-the-shelf object detector and reduce its inference latency on video data by large margins with minimal sacrificing of accuracy. We demonstrated that MOVEX improves performance for existing object detection models, for which online real-time video object detection would not have been possible prior. Additionally, we showed that accuracy improvements are possible without sacrificing latency through increasing the resolutions of the models and using these models with MOVEX. Lastly, MOVEX allows for models typically restricted to the domain of GPU or TPU computing due to latency concerns to expand to less expensive CPU devices.

There are several opportunities for further improvements to this technique. Further parallelism improvements are possible within the optimistic sparse detection propagation function since the application of the function to a detection is independent of the other detections, allowing for updating multiple detections simultaneously. This would allow for further latency reduction but would add a nonnegotiable dependency on having a GPU, as the number of detections could be large.

## Figures and Tables

**Table 1:** Typical resolutions for associated numbers of optical flow vectors and motion vectors.

Image Resolution	Dense Flow Vectors	16 × 16 MVs
720×1280	9.216 × $10^{5}$	3600
1080×1920	$2.0736\times 10^{6}$	8100
3840×2160	$8.2944\times 10^{6}$	32,400

**Table 2:** Evaluation of MOVEX with H.264 MVs or FlowNet2 optical flow [9] against baseline Faster R-CNN model across both MOT20 [14] and MOT16 [30] datasets. Additionally, two YOLOv4 models [1] trained on the COCO dataset [17] with varied input resolutions (416×416 and 960×960) demonstrate the accuracy gains possible without sacrificing inference latency when using the MOVEX technique.

Method	Dataset	Avg Latency (ms) ↓	AP ↑
FRCNN [14]	MOT20	131.52	0.63
FRCNN [14] with MOVEX + FlowNet2	MOT20	259.17	0.629
FRCNN [14] with MOVEX + H.264 MVs	MOT20	13.81	0.625
YOLOv4 [1] (960 × 960)	MOT20	79.46	0.405
YOLOv4 [1] (960 × 960)	MOT20	11.73	0.404
with MOVEX + H.264 MVs			
YOLOv4 [1] (416 × 416)	MOT20	26.34	0.261
YOLOv4 [1] (416 × 416) on CPU	MOT20	190.41	0.261
YOLOv4 [1] (416 × 416) on CPU	MOT20	7.96	0.257
with MOVEX + H.264 MVs			
FRCNN [33]	MOT16	130.22	0.688
FRCNN [33] with MOVEX + FlowNet2	MOT16	198.38	0.527
FRCNN [33] with MOVEX + H.264 MVs	MOT16	4.78	0.546
YOLOv4 [1] (960 × 960)	MOT16	81.80	0.672
YOLOv4 [1] (960 × 960)	MOT16	4.14	0.59
with MOVEX + H.264 MVs			
YOLOv4 [1] (416 × 416)	MOT16	28.26	0.455
YOLOv4 [1] (416 × 416) on CPU	MOT16	215.28	0.455
YOLOv4 [1] (416 × 416) on CPU	MOT16	3.76	0.34
with MOVEX + H.264 MVs			

Downwards arrow depicts lower is better, while upwards arrow depicts higher is better.

**Table 3:** Comparison between using global movement compensation in addition to local aggregations versus only local aggregation.

Method	Dataset	Avg Latency (ms)	AP
Proposed	MOT20 [14]	13.81	0.626
Without Global Comp	MOT20 [14]	13.17	0.582
Proposed	MOT16 [30]	4.78	0.546
Without Global Comp	MOT16 [30]	4.46	0.505

**Table 4:** Comparison between traditional single-process approaches versus multi-process-based optimistic sparse detection propagation.

Method	Dataset	Avg Latency (ms)	AP
Proposed	MOT20 [14]	13.81	0.626
Fifth Frame	MOT20 [14]	42.75	0.619
Tenth Frame	MOT20 [14]	29.46	0.501
Proposed	MOT16 [30]	4.78	0.546
Fifth Frame	MOT16 [30]	33.60	0.519
Tenth Frame	MOT16 [30]	19.43	0.426

**Table 5:** Comparison between using the median as an aggregation function versus perturbing based on the center motion vector in a given detection.

Method	Dataset	Avg Latency (ms)	AP
Proposed (Median)	MOT20 [14]	13.81	0.626
Center Sample	MOT20 [14]	15.63	0.623
Proposed (Median)	MOT16 [30]	4.78	0.546
Center Sample	MOT16 [30]	5.21	0.54

**Table 6:** Comparison between detections not using any perturbation versus with perturbation.

Method	Dataset	Avg Latency (ms)	AP
Proposed	MOT20 [14]	13.81	0.626
No Perturbation	MOT20 [14]	1.07	0.618
Proposed	MOT16 [30]	4.78	0.546
No Perturbation	MOT16 [30]	0.63	0.501

**Table 7:** Performance comparison between various encoding qualities. The proposed results made use of the high quality encoder settings.

Method	Dataset	Avg Latency (ms)	AP
Proposed	MOT20 [14]	13.81	0.626
Medium Quality	MOT20 [14]	16.19	0.625
Low Quality	MOT20 [14]	14.66	0.625
Proposed	MOT16 [30]	4.78	0.546
Medium Quality	MOT16 [30]	5.47	0.509
Low Quality	MOT16 [30]	5.30	0.509

## Data Availability

Publicly available datasets were analyzed in this study. This data can be found here: https://motchallenge.net/ (accessed on 11 May 2023).

## References

[1] Bochkovskiy A., Wang C.Y., Liao H.Y.M. (2020). YOLOv4: Optimal Speed and Accuracy of Object Detection. arXiv.

[2] Redmon J., Farhadi A. (2018). YOLOv3: An Incremental Improvement. arXiv.

[3] Ren S., He K., Girshick R., Sun J. (2017). Faster R-CNN: Towards Real-Time Object Detection with Region Proposal Networks. IEEE Trans. Pattern Anal. Mach. Intell..

[4] Liu W., Anguelov D., Erhan D., Szegedy C., Reed S., Fu C.Y., Berg A. SSD: Single Shot MultiBox Detector. Proceedings of the Computer Vision—ECCV 2016: 14th European Conference.

[5] Park H.J., Kang J.W., Kim B.G. (2023). ssFPN: Scale Sequence (S 2) Feature-Based Feature Pyramid Network for Object Detection. Sensors.

[6] Wang C.Y., Bochkovskiy A., Liao H.Y.M. YOLOv7: Trainable bag-of-freebies sets new state-of-the-art for real-time object detectors. Proceedings of the IEEE/CVF Conference on Computer Vision and Pattern Recognition.

[7] Pandey V., Anand K., Kalra A., Gupta A., Roy P.P., Kim B.G. (2022). Enhancing object detection in aerial images. Math. Biosci. Eng..

[8] Mao H., Yang X., Dally W.J. A delay metric for video object detection: What average precision fails to tell. Proceedings of the IEEE/CVF International Conference on Computer Vision.

[9] Ilg E., Mayer N., Saikia T., Keuper M., Dosovitskiy A., Brox T. FlowNet 2.0: Evolution of Optical Flow Estimation with Deep Networks. Proceedings of the 2017 IEEE Conference on Computer Vision and Pattern Recognition (CVPR).

[10] Dosovitskiy A., Fischer P., Ilg E., Häusser P., Hazirbas C., Golkov V.v.d., Smagt P., Cremers D., Brox T. FlowNet: Learning Optical Flow with Convolutional Networks. Proceedings of the 2015 IEEE International Conference on Computer Vision (ICCV).

[11] Zhu X., Dai J., Yuan L., Wei Y. Towards High Performance Video Object Detection. Proceedings of the 2018 IEEE/CVF Conference on Computer Vision and Pattern Recognition.

[12] Zhu X., Xiong Y., Dai J., Yuan L., Wei Y. Deep Feature Flow for Video Recognition. Proceedings of the 2017 IEEE Conference on Computer Vision and Pattern Recognition (CVPR).

[13] Leal-Taixé L., Milan A., Reid I., Roth S. (2015). MOTChallenge 2015: Towards a Benchmark for Multi-Target Tracking. arXiv.

[14] Dendorfer P., Rezatofighi H., Milan A., Shi J., Cremers D., Reid I., Roth S., Schindler K., Leal-Taixé L. (2020). MOT20: A benchmark for multi object tracking in crowded scenes. arXiv.

[15] Krebs S., Duraisamy B., Flohr F. A survey on leveraging deep neural networks for object tracking. Proceedings of the 2017 IEEE 20th International Conference on Intelligent Transportation Systems (ITSC).

[16] Everingham M., Van Gool L., Williams C.K.I., Winn J., Zisserman A. (2010). The Pascal Visual Object Classes (VOC) Challenge. Int. J. Comput. Vis..

[17] Lin T.Y., Maire M., Belongie S., Hays J., Perona P., Ramanan D., Dollár P., Zitnick C.L., Fleet D., Pajdla T., Schiele B., Tuytelaars T. (2014). Microsoft COCO: Common Objects in Context. Proceedings of the Computer Vision—ECCV 2014, Zurich, Switzerland, 6–12 September 2014.

[18] Girshick R. Fast R-CNN. Proceedings of the 2015 IEEE International Conference on Computer Vision (ICCV).

[19] Redmon J., Divvala S., Girshick R., Farhadi A. You only look once: Unified, real-time object detection. Proceedings of the IEEE Conference on Computer Vision and Pattern Recognition.

[20] Wang C.Y., Liao H.Y.M., Wu Y.H., Chen P.Y., Hsieh J.W., Yeh I.H. CSPNet: A New Backbone That Can Enhance Learning Capability of CNN. Proceedings of the IEEE/CVF Conference on Computer Vision and Pattern Recognition (CVPR) Workshops.

[21] He K., Zhang X., Ren S., Sun J. (2015). Spatial Pyramid Pooling in Deep Convolutional Networks for Visual Recognition. IEEE Trans. Pattern Anal. Mach. Intell..

[22] Liu S., Qi L., Qin H., Shi J., Jia J. Path Aggregation Network for Instance Segmentation. Proceedings of the IEEE Conference on Computer Vision and Pattern Recognition (CVPR).

[23] Ujiie T., Hiromoto M., Sato T. Interpolation-Based Object Detection Using Motion Vectors for Embedded Real-Time Tracking Systems. Proceedings of the IEEE Conference on Computer Vision and Pattern Recognition (CVPR) Workshops.

[24] Liu Q., Liu B., Wu Y., Li W., Yu N. (2019). Real-Time Online Multi-Object Tracking in Compressed Domain. IEEE Access.

[25] Tabani H., Fusi M., Kosmidis L., Abella J., Cazorla F.J. IntPred: Flexible, Fast, and Accurate Object Detection for Autonomous Driving Systems. Proceedings of the 35th Annual ACM Symposium on Applied Computing, SAC’20.

[26] He K., Zhang X., Ren S., Sun J. Deep Residual Learning for Image Recognition. Proceedings of the 2016 IEEE Conference on Computer Vision and Pattern Recognition (CVPR).

[27] Dai J., Li Y., He K., Sun J. R-FCN: Object Detection via Region-Based Fully Convolutional Networks. Proceedings of the 30th International Conference on Neural Information Processing Systems, NIPS’16.

[28] JV Team Draft ITU-T Recommendation and Final Draft International Standard of Joint Video Specification. ITU-T Rec. H.264 2003. https://www.itu.int/wftp3/av-arch/jvt-site/2003_09_SanDiego/JVT-I023r1.doc.

[29] You W., Sabirin H., Kim M. Moving object tracking in H.264/AVC bitstream. Proceedings of the Multimedia Content Analysis and Mining: International Workshop, MCAM 2007.

[30] Milan A., Leal-Taixe L., Reid I., Roth S., Schindler K. (2016). MOT16: A Benchmark for Multi-Object Tracking. arXiv.

[31] Menze M., Heipke C., Geiger A. (2018). Object Scene Flow. ISPRS J. Photogramm. Remote Sens. (JPRS).

[32] Menze M., Heipke C., Geiger A. (2015). Joint 3D Estimation of Vehicles and Scene Flow. ISPRS Ann. Photogramm. Remote Sens. Spat. Inf. Sci..

[33] Yu F., Li W., Li Q., Liu Y., Shi X., Yan J., Hua G., Jégou H. (2016). POI: Multiple Object Tracking with High Performance Detection and Appearance Feature. Proceedings of the Computer Vision—ECCV 2016 Workshops, Amsterdam, The Netherlands, 8–16 October 2016.

[34] FFMPEG Documentation. https://ffmpeg.org/ffmpeg.html.

[35] Butler D.J., Wulff J., Stanley G.B., Black M.J. (2012). A naturalistic open source movie for optical flow evaluation. Proceedings of the European Conference on Computer Vision (ECCV), Florence, Italy, 7–13 October 2012.

